# ENHANCING GERONTOLOGY EDUCATION: AN HISTORICAL PERSPECTIVE

**DOI:** 10.1093/geroni/igae098.0816

**Published:** 2024-12-31

**Authors:** Harvey Sterns

**Affiliations:** University of Akron, Akron, Ohio, United States

## Abstract

Beginning in 1972 with support from the Administration on Aging (AoA), the Ad Hoc Committee on Development of Gerontology Resources was formed to mobilize gerontological resources in response to the Administration on Aging’s proposal for the establishment and funding of regional gerontology centers. A major aspect of this initiative was to provide educational opportunities at all levels. This effort was based on the anticipated need for personnel to serve the present and future number of older adults. The work of this committee led to the formation of the Association for Gerontology in Higher Education (AGHE) with a formal affiliation with the Gerontological Society in 1973. Programs were supported by AoA to develop undergraduate and graduate certificates with degrees in disciplines related to aging; associate, bachelor, and master’s degrees in Gerontology; and minors in Gerontology. This early funding played an important role in colleges and universities developing programs in gerontology education. Over the last 50 years, important work has been done to optimize the development of gerontology education, including the AGHE Standards and Guidelines and the AGHE Gerontology Competencies for Undergraduate and Graduate Education that serve as the basis for accrediting gerontology degree programs through the Accreditation for Gerontology Education Council. The need for gerontology education programs is even greater today given the shortage of personnel in all areas of gerontological services. However, the number of programs has grown slowly with increasing concern about how to maintain support for existing programs and find new funding to develop new programs.

